# When Push Comes to Shove—The Moral Fiction of Reason-Based Situational Control and the Embodied Nature of Judgment

**DOI:** 10.3389/fpsyg.2020.00203

**Published:** 2020-02-14

**Authors:** Lasse T. Bergmann, Jennifer Wagner

**Affiliations:** Institute of Cognitive Science, Osnabrück University, Osnabrück, Germany

**Keywords:** moral cognition, embodied cognition, enactive account of perception, moral judgments, social intuitionism

## Abstract

It is a common socio-moral practice to appeal to reasons as a guiding force for one’s actions. However, it is an intriguing possibility that this practice is based on fiction: reasons cannot or do not motivate the majority of actions—especially moral ones. Rather, pre-reflective evaluative processes are likely responsible for moral actions. Such a view faces two major challenges: (i) pre-reflective judgments are commonly thought of as inflexible in nature, and thus they cannot be the cause of the varied judgments people rely on in everyday life, and (ii) if reflective reason-based judgments do not play a strong causative role in judgment, why do people rely on the articulation of reasons in their moral practices? And how is moral agency and moral theorizing possible without it? We argue that the pre-reflective judgments motivating moral actions are embodied in nature. The experience of the rightness of an action that drives a person to act depends on the sensorimotor interactions that have cultivated an agent’s perspective on the world. These interactions are embedded in relational contexts, relative to which judgments are individuated. Because of this relational embeddedness, they are more flexible than they are commonly thought to be, enabling us to explain the variety of human behavior by appealing to them. The Anglo-European practice of appealing to reason as if they were propositional belief-statements motivating actions can be accounted for as nothing more than an idiosyncratic way of constructing narratives to clarify and express the relational context of intentional actions.

## Introduction

Since the Enlightenment, our understanding of what it means to be human is marked by a sharp division into mind and body. Doris and Nichols (2012, p. 425) argue that “much of the work in contemporary [sic!] cognitive science and moral philosophy is Cartesian in spirit: it appears to presuppose that human beings reason best on their own, windows closed and curtains drawn, after the gripping fiction of Descartes’ Meditations.” For all the good the Enlightenment brought to the western world, it also may have entrenched some questionable assumptions. For when the Enlightenment succeeded in establishing the superiority of reason as basis for moral conduct, it simultaneously threw, as MacIntyre aptly put it, “the language and practice of morality into grave disorder” (MacIntyre, 1981, 3).

This cartesian division is much more than a simple split into mind and body, it has become a systematic division of two realms of mental activity (cf. Jaggar, 1989) pervading everything from folk psychology to academic discourse: Cognition vs. Emotion, Reason vs. Affect, Objective vs. Subjective, etc. In contemporary psychology, we see this division most clearly in dual process theories of the mind (cf. Kahneman et al., 1982; Greene et al., 2001, 2009; Sunstein, 2005; Kahneman, 2011; Greene, 2014). These theories posit two modes of cognition, one based on reason, control, and rational agency, and one based on emotion, automaticity, and external determination. It is, of course, a fallacy to treat all dual process theories with one broad stroke (Evans, 2012), but a more nuanced discussion is beyond the scope here. Section “Where Is the Neuropsychological Pathway Facilitating Reason-Based Action Control?” deals with some of the evidence opposing the view that neural areas identified with rational agency are primarily involved in moral actions.

The critical intuition we oppose in this paper is that human moral conduct can only be based on the exercise of cognitive control (e.g., Piaget, 1932/1965; Kohlberg, 1969, 1976; Turiel, 1983). We believe that this intuition is based on multiple assumptions, which are questionable (c.f. Haidt, 2001). The first issue we will take up in the following is whether we usually act for reasons. Because we can articulate reasons for our action, we commonly assume that there are mental states preceding our actions which we can consciously reflect upon, which determine our actions. These mental states are propositional or can at least be articulated propositionally. We believe that there is good evidence to doubt that such reflectively endorsed (e.g., Haidt, 2001), conscious, propositional mental states have the power to control most of our actions.

It is, however, not necessary to reject reason-based cognitive control all together to appreciate that many actions are not based on this control. The main part of our argument is that *most* moral actions are embodied and not based on reason. While moral actions have occasionally been recognized to be embodied (cf. Prinz, 2006; Gigerenzer, 2008; Greene et al., 2009; Francis et al., 2016), this has often been interpreted as a deficiency. We argue that embodied moral actions are flexible enough to explain the diversity of human moral behavior and do allow for meaningful moral analysis.

The flexibility of embodied moral actions has been doubted so far. Greene (2014, p. 148), for example, says: “And our brains have a manual mode, a general capacity for conscious, explicit, practical reasoning that makes human decision making flexible.” Even Gigerenzer (2008) who maybe presents the most charitable account of pre-reflective moral judgments does not recognize them to be flexible. In his account, they are evolutionary adaptations, that work well in moral context that we are evolved to deal with or exploit. Gigarenzer also seemingly recognizes the embodied nature of moral judgments, but his notion of embodiment is not the same as the one we will utilize in the following. Gigarenzer presents one of the most insightful accounts of moral intuitions, yet he is still limited by his computational perspective. We will leave this orthodox approach to cognitive science behind, and embrace a truly embodied framework to understand reasons. The “situated turn” in cognitive science (Varela et al., 1991; Damasio, 1994; Clark and Chalmers, 1998; Lakoff and Johnson, 1999; Noë, 2004; Prinz J., 2004; Prinz J.J., 2004; Colombetti, 2007; Colombetti and Thompson, 2008; Stephan et al., 2014, etc.) provides a much more substantial theoretical framework, that actually can deliver on what Gigerenzer (2008, p. 10–11) rightly identified as flaws in psychological research into the moral mind: Their sole focus on individual reasoning, artificial moral problems, and self-reports, as opposed to interactions and groups dynamics, the study of everyday moral problems in the wild, and a focus moral actions. We argue that moral actions are strongly embodied and pre-reflective evaluations implicit in actions (embodied judgments) are tied to features of the bodily interaction of an agent with the world. Their flexibility derives from the relational specificity of these embodied judgments, that we have specific repertoires of interaction possibilities in specific relational contexts, and, thus the concrete occurrence of an embodied judgment depends on how an agent relates to a specific state of affairs, as well as which embodied judgments this agent has cultivated in this specific relational context.

Finally, we challenge the idea that moral philosophy necessarily relies on the analysis of reasons for actions. Clearly, it is somewhat of the standard operating procedure for many ethicists to do so, yet there are many approaches to moral philosophy which rely on different kinds of ethical analysis. MacIntyre (1981) demonstrates that this kind of analysis is a product of the Enlightenment, favoring a Thomist approach to ethics himself. But there are also other approaches to ethics, that favor the analysis of relationship over individual’s mental states (e.g., Noddings, 1984; Gilligan, 1993; Held, 2006, etc.) or experiential and narrative features (e.g., Levinas, 1969; MacIntyre, 1981; Ricoeur, 1992). We understand the contribution cognitive science can make to ethical discourse as providing boundaries for moral theories: Many ethical theories make implicit or explicit assumptions about how humans act and experience the world, which are descriptive and not normative in spirit and thus can be more or less plausible in light of experimental evidence.

## Reasons, Motivation, and Action

**FIGURE 1:**
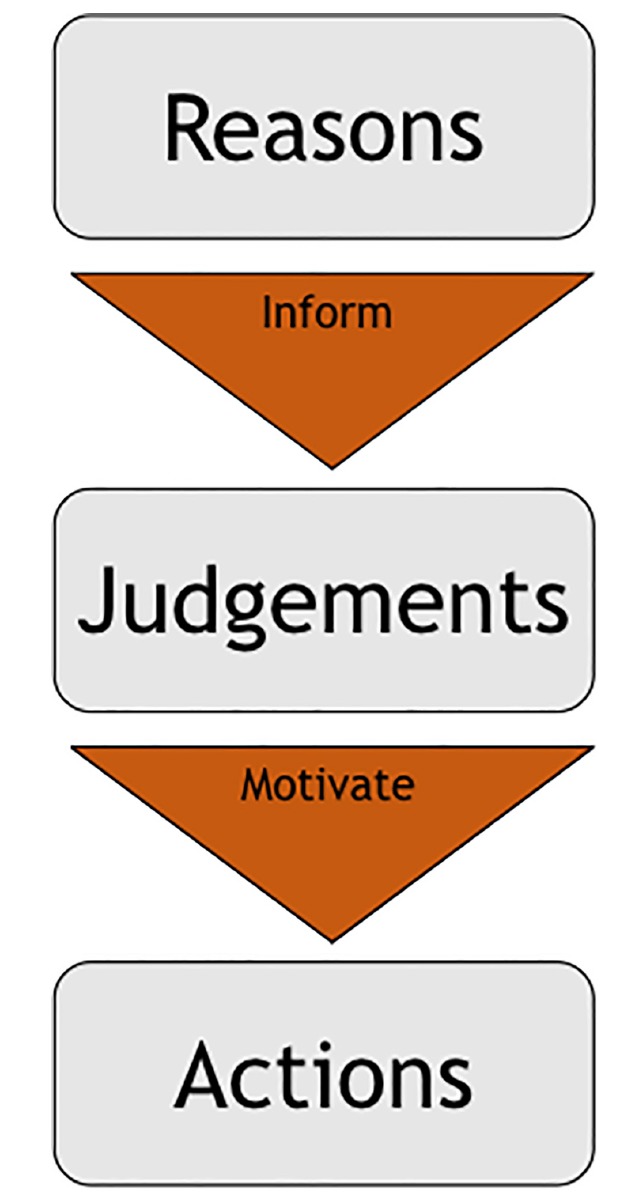
Rationalist account of moral action.

The everyday experiences of weighing reasons for different paths of future action or articulating reasons for past ones appear too persuading to just dismiss. If factors that motivated behavior irrespective of reasons are identified, those are usually dismissed as biases or automatic reactions. This intuition is also dominant in contemporary research on moral cognition, which is built on the assumptions of orthodox cognitivism: “a rationalist model in which emotions and body states may be taken into account by reason but it is pure reasoning that ultimately leads to moral decisions” (Wilson and Foglia, 2017). We will first argue that reasons are mostly not motivational, so they have little to do with the concrete judgments that bring about action. After we explore the nature of these judgments, we will return to the role of reasons; we will argue that, although they are not motivational, reasons play a critical role in moral conduct (Figure 1).

### Unreflected Processes Undermining Reason

Psychological studies in the last couple of decades have called the veracity of articulated reasons as causes of action into question. For while people are competent in articulating reasons for their actions, these articulated reasons often played no part in bringing said actions about.

Todorov et al. (2005) were able to predict the choice of candidates in an election in over 75% of cases, solely based on participants’ preferences for the faces of the candidates. While people usually elaborate detailed reasons why they prefer one candidate over the other, in three out of four cases, the outward appearance predicted their preferences. A number of related studies since then have found similar effects (c.f. Walter, 2016, Ch. 12) in the United States (Ballew and Todorov, 2007; Benjamin and Shapiro, 2009), Switzerland (Antonakis and Dalgas, 2009), Australia (King and Leigh, 2009), Germany (Rosar et al., 2008), and many others. These results suggest that the participants might not always have reliable access to the factors informing their decision.

Johansson et al. (2005, 2006) made similar observations. They showed participants two photos depicting similar-looking women and asked them to indicate which was more attractive. Subjects were then supposedly handed the picture they chose, but the experimenter stealthily switched the pictures so that the participant ended up with the picture of the person they had judged to be less attractive. The participants rarely noticed this deception. When prompted to give reasons for why they chose the person in the picture over the other one they articulated reasons that applied not to the person they had actually chosen but to the person in the picture they were holding. While the participants might very well have been convinced that they were presenting the reasons on which they based their judgment, this could not have been the case.

Hofer (2015) recruited 780 teachers from German-speaking countries to assign grades to answers which they were told were given by students in physics class. The answers themselves were identical across conditions, the only difference was the gender of the student who supposedly gave the answer—the content of the answer itself was identical. Yet, the study reports that teacher’s assessments varied by up to a whole grade depending on the gender of the student, the gender of the teacher and the teacher’s experience. It is highly unlikely that this bias in grading is the product of reflected conscious choice. Instead, the reference to the student’s gender infiltrates the mind and shapes behavior in unconscious, unwanted ways.

Ahis sensorimotor componesnother case is presented by Darley and Batson (1973), who isolate situational factors undermining the power of reflected choice.^1^ Theology students in a seminary school were told to prepare a talk and then go to another building to deliver it. The participants were given different information on whether or not they were on time. On the way to their talk, they encountered a man in need of medical attention. As it turns out neither the character of the student nor the content of the talk, they were preparing had an effect on their willingness to help. But those who had time to spare helped in 63% of cases, while those already too late only helped in 10% of cases. It is noteworthy that the preparation of the talk in one of the variations of the experiments required the student to reflect on the story of the Good Samaritan from the bible, but had no effect on the behavior of the participants. Participants stated that time pressure, which may have been a plausible if morally questionable reason for their actions, was not a factor that influenced their decisions. These findings suggest that even though agents generally experience themselves to act solely on the basis of reasons, in many cases, this proves to be an illusion.

Perhaps the most famous findings contradicting the first claim were provided by Haidt et al. (2000; cf. Haidt, 2001). In their study, they presented participants with thorny moral problems. Because these problems were carefully constructed, the reasons that generally apply in situations of the same kind did not apply in the concrete scenario at hand. When participants were asked to explain the judgments they had formed, they went through a process of identifying a reason which would usually be applicable, but quickly realize that the reason did not actually sensibly apply to the scenario they were facing (Haidt, 2001, 1024). They moved on to the next reason, only to find that it did not apply to the particular situation either. Finally, they gave up on trying to find adequate reasons to justify their judgment.

### Where Is the Neuropsychological Pathway Facilitating Reason-Based Action Control?

The assertion made by rationalists is that reasons motivate and control behavior; however, there seems to be no plausible neural pathway that would facilitate such control. As Schroeder (2004) points out, affective-motivational structures (i.e., non reason-based processes) in the ventromedial prefrontal cortex (vmPFC)^2^ are involved in any kind of supposed reflective or reason-based action control. This means that the motivational force of a reason is mediated or overridden by the vmPFC, where they come into conflict with or are overridden by other motivational processes. The problem this poses for rationalists—besides the dreaded involvement of affectivity—is that the motivational power of the vmPFC may be independent of reasons even if reasons are an indirect causal precursor. The vmPFC is sensitive to learned rewards (Schroeder, 2004, 147–148) and not to the nuances of a reflected reason. Even if a conscious reflected belief specified a certain way in which an action should be controlled, this mental content would be lost along the neural path (as will become apparent in the following paragraphs).

To illustrate this, one may imagine the mind like a set of mirrors and filters that change the composition of an incoming picture to produce a specific output. The part of the mirror system that represents reasons produces a colorful picture, but at some point, it passes through a filter which transforms the picture into a black and white image. Through this transformation, information is lost, not all of course, but in this case, the important bits. This filter is the neural translation of signals into the embodied representations of the vmPFC. Here, the nuances of reasons encoded in the color spectrum are lost, and new, different colors are imposed on the picture. The sleek linguistic encodings of reasons have made way for encodings of bodily movement. Looking at the picture, only vague instructions still resemble the preceding reasons, recognizable as “do *something*” or “careful.”

Greene (2014) posits a theory of dual-process morality, where there is a competition between rational and affective processes, claiming that the motivational superiority of the vmPFC is not a forgone conclusion, but controlled-reflected reasons may not be overridden by the vmPFC as long as people are not emotionally engaged. However, it seems doubtful that any sort of real-life moral task could be void of affective processes. The alternative pathway suggested by Greene involving the dorsolateral prefrontal cortex is unsupported by the meta-analysis of Eres et al. (2018), which furthermore shows consistent involvement of the vmPFC in moral cognition tasks. This may mean that there is no neurophysiological pathway that would allow consciously reflected, propositional content, i.e., reasons, to motivate actions directly.

## What Is the Motivational Component in Moral Actions?

The strongest argument against reason-based action control is that judgments motivating actions are not based on reason, but are embodied in nature. Initial evidence for this embodiment claim is supplied by Greene et al. (2009), in their investigation of Trolley cases (cf. Foot, 1967; Thomson, 1976, 1984). The basic idea of these thought experiments is that a person finds themselves faced with a problematic decision: they can take action to save a number of people (usually five) at the expense of another person or do nothing. The two most prominent variants of this dilemma are the lever variant and the footbridge variant. In the *lever variant*, a runaway trolley—after which the experiment is named—barrels down a track toward five railway workers unable to escape it. However, one can redirect the trolley to another track by pulling a lever. On this alternative track is a single railway worker which would be hit by the trolley. The usual intuition in this case is that one should pull the lever.^3^ In the *footbridge variant*, there are a few crucial differences. The trolley cannot be redirected, but it can be stopped. To stop it, one would have to push a large person off a footbridge arching over the track. People’s intuitions in this case are usually different from the first case; the majority of people would be very reluctant to push the large person off the footbridge.

Given the rationalist model of moral decision making, these results indicate that—even though the scenarios might initially seem the same—there are different reasons applicable to the lever and the footbridge case, respectively. For a long time, moral philosophers have debated what these reasons might be. Moral psychology, then, brought a different approach (and substantial normative baggage, cf. Bergmann, 2019) to the affair, nicely summed up by Greene: “Trolley dilemmas are useful, not because they are representative, but because they are artificial high-contrast stimuli that enable us to dissociate cognitive processes that are otherwise hard to dissociate” (Greene, 2015, 10, cf. Cushman and Greene, 2012). Trolley problems are no longer employed to generate ethical insight, but rather to generate insights into the different cognitive processes at play. To figure out what differentiates the footbridge from the lever variant, Greene et al. (2009) introduce a further case, the *trapdoor variant*, in which one does not push the large person onto the tracks, but instead pulls a lever to drop them through a trapdoor. Participants are significantly more comfortable opening the trapdoor than pushing the large person. The difference in judgment, then, seems to stem from one major factor “primarily moderated by whether the outcome is brought about by personal contact, which typically involves the use of personal force” (Feltz and May, 2017, 314). This factor was the only(!) stable one, in a meta analysis compiling 101 experiments with over 24,000 participants (Feltz and May, 2017).

Greene et al. (2009, 370) sum up their findings regarding the personal force factor as follows: “In a general sense, [this result] suggests a mechanism of moral judgement that is a species of embodied cognition (Lakoff and Johnson, 1999; Prinz, 2002; Wilson, 2002; Gallese et al., 2004; Prinz J., 2004; Prinz J.J., 2004). One natural source of such embodied goal representations is [the] system of action planning […]” However, if moral judgments rely on such embodied representations, then they appear to be modality specific or non-propositional in content. Either of these possibilities would be problematic for reason-based action control as reasons are usually propositional and modality neutral. So by the neural translation of reasons into modality specific and non-propositional representations, they are in danger of losing their specific content.

While Greene only tentatively raises the possibility of an embodied process, these speculations are substantiated in studies by Francis et al. (2016, 2017). These studies varied the involvement of the body in the footbridge case utilizing a virtual reality environment. In a first experiment, participants were presented the footbridge scenario in the VR environment and then asked which decision they would make via a text-prompt. Contrary to usual experimental findings, participants mostly chose to push the person off the footbridge (Francis et al., 2016). In a follow-up experiment, participants were no longer asked to make a choice via a text-prompt, but actually perform a bodily action, i.e., move a joystick or shove a mannequin to push the person in the simulation off the footbridge (Francis et al., 2017). It turns out that participants judged differently depending on how much involvement of the body the experiment was set up for. More involvement of the body triggered the characteristic personal force factor, implying that it is indeed an embodied process.

As we have seen in the analysis of the trapdoor case and Francis et al.’s VR studies at least some moral actions are embodied, in the following we set out to give context to this embodiment claim and flesh out how specific embodied judgments are cultivated. We will not commit to a strong distinction between social and moral cognitive processes (cf. Gigerenzer, 2008, p. 9–10; O’Neill, 2017) and thus discuss evidence we think is concerned with broadly evaluative cognitive processes. For the time being, we will set aside questions regarding the justification, representational nature, and conscious accessibility of embodied moral judgments, focusing solely on how they come about. We especially aim to illuminate why certain circumstances invite different embodied judgments. Regarding the difference between the trapdoor and the footbridge case, one strategy would be to appeal to the experiential difference between the two cases: Levers are experienced as pullable, but people are not experienced as pushable. The question is why these scenarios offer different affordances. In the philosophical study of experience, the body plays a central role, as exemplified by Husserl (1912/1989); Merleau-Ponty (1945/1962), and Levinas (1969).^4^ While we draw on these sources in spirit, we will mostly rely on more empirically motivated accounts of embodiment.

We will first explore how embodied evaluative processes depend on specific bodily capacities and cultivation. The most relevant thesis of embodiment for moral cognition is Casasanto’s (2009, 351) body-specificity-thesis, which postulates that “people with different bodily characteristics, who interact with their physical environments in systematically different ways, should form correspondingly different mental representations.” In other words, there should be cognitive processes, which are determined and constrained by the continued interactions of agent’s bodies with their specific environment (Casasanto, 2011). Casasanto (Willems et al., 2010) provides evidence that action verbs like “throwing” and “grasping” are differently neurally lateralized in motor areas depending on whether participants are right- or left-handed. Neural lateralization, i.e., the brain hemisphere in which functions are localized, is not fixed at birth. Rather, brains are malleable through experience, and this research shows that they are molded depending on specific bodily capacities. The normative valence of perception, i.e., the sense of appropriateness we experience as part of every perception, is equivalently molded by bodily capacities. In a further study, Casasanto (2009) asked participants to draw an object on the right or left side of a piece of sketch-paper, after they read a story that framed the object as good or bad. Left-handed participants drew a “good” animal on the left side in 74% of cases and right-handed participants drew the “good” animal on the right side in 67% of cases. In horizontal control trials, both left- and right-handed participants strongly preferred the upper half for the “good” animal. This effect also occurs when participants had to say on which side of a sketch paper an animal should be placed without drawing it themselves. Casasanto (2009) provides one further generalization of this effect. Subjects were also asked to evaluate alien creatures. If the alien was presented on the dominant side of the person, it was perceived more positively and more negatively when presented on the non-dominant side. This result suggests that judgments are partly dependent on motor capacities. Casasanto further investigates the body-specificity-thesis in the area of motivation, i.e., whether specific action motivation depends on hand dominance. Brookshire and Casasanto (2012) present evidence that lateralization of avoidance and approach motivation depends on whether participants are right or left-handed, in support of the sword and shield hypothesis. The idea is that participants learn to approach objects with their strong side, which is more capable of interacting with objects, and turn their weak side toward objects to be avoided, because damage to this side is less incapacitating (cf. Casasanto, 2014). These insights lead us to formulate a sort of body-specificity-thesis of moral judgment.

The idea is that humans learn motor competencies, i.e., ways in which to move their bodies. There are innumerable ways to perform a specific action, but throughout people’s lives, they develop a specific style of movement. Young (1980) explores the feminine experience and expressiveness of movement. Aptly, she named her seminal paper “Throwing Like a Girl.” It is stereotypical, but still too often accurate, that there are gender-specific styles of movement. While male and female bodies differ, these differences do not explain the different styles of movement, though, Young is forced to mockingly refute Strauss on this point: “He is somewhat at a loss, however, to specify the source of the difference. Since the feminine style of throwing is observed in young children, it cannot result from the development of the breast.” It is evident that women with female bodies can throw in a way that is stereotypically manly and men with male bodies can throw “like a girl.” Thus, the specificity of bodily movement is likely due to cultural forces. People bow to these forces, because they have become ingrained in their experience of movement. As Young puts it in regard of female styles of movement:

“The objectifying regard which ‘keeps her in her place’ can also account for the spatial modality of being positioned and for why women frequently tend not to move openly, keeping their limbs enclosed around themselves. To open her body in free active and open extension and bold outward directedness is for a woman to invite objectification.”

Certainly, this explanation does not indicate the content of the experience of movement. It simply feels wrong to move one’s body in certain ways, much like it may feel wrong to see a contortionist twist their body.^5^

Our proposal is that these styles or motor patterns are judgments, i.e., that in them an evaluation is implicit. People are taught from an early age how they should and should not use their bodies. A lever is pullable, as levers are usually designed and placed to be used. When humans learn to use them, they develop motor patterns how to interact with levers and the interactions become comfortable. However, a person is not pushable because when children experiment with this motor pattern on the playground they are taught not to use their bodies in this way. Strictly speaking, they may have the capacity to push people, but it is dormant—experienced as impermissible. The difference of perceived permissibility, of motivational pull, encountered in the variants of the Trolley-problem, can be explained this way. Thus, such explanations merit further study.

However, embodied judgments of this kind initially seem to constitute all-or-nothing norms. If judgments are based on learned motor capacities, then two identical physical interactions should elicit the same judgment. However, especially in interpersonal situations, there are ways of interacting that are permissible in some situational and relational context but not in others. It is, for example, usually wrong to kiss a stranger, but it is often acceptable to kiss one’s partner. Though these actions rely on the same motor pattern, it is clear that they are experienced differently. People implicitly judge it to be appropriate and are motivated to kiss their partners, but not random people on the street. So, at first glance, the embodied judgments discussed here lack flexibility, the precise charge often leveled against non-reason-based judgments. The next section will be concerned with understanding the relational embeddedness of embodied judgments which is necessary to account for the flexibility normative experiences made, especially in interpersonal relationships.

## Relational Specificity of Motor Patterns

In their seminal text, *The Embodied Mind*, Varela et al. (1991, 172–173) explicate their notion of embodiment:

“By using the term embodied, we mean to highlight two points: first, that cognition depends upon the kinds of experience that come from having a body with various sensorimotor capacities, and second, that these individual sensorimotor capacities are themselves embedded in a more encompassing biological, psychological, and cultural context.”

This sensorimotor component of enactive theory draws upon the phenomenology of Merleau-Ponty (and Husserl). This tradition also served as an inspiration for Hubert dreyfus, an ardent critic of orthodox cognitive science and a staunch proponent of integrating phenomenological accounts of cognition with cognitive science. Dreyfus and Dreyfus (1991) provide an account of motor-expertise, a form of practical knowledge, which agents acquire in stages through continued interaction with the world. Similarly, O’Regan and Noë (2001) provide a sensorimotor theory of experience, arguing that conscious experience relies on agents’ (acquired) implicit grasp of sensorimotor dependencies. Both accounts are designed to make these theories palatable for orthodox cognitive science and are, thus, overly attached to concepts like reflection, choice, and reasons, which is problematic for the line of thought advanced in this text. However, the core of their ideas is applicable.

### Styles of Movement as Embodied Judgments

What we want to highlight is that agents acquire “styles” of interaction with the world that define their relationship with it.^6^ Constrained by the capacities of their bodies and the physical structure of the world, there emerges a theoretical space of action possibilities. Throughout their lives, people navigate the world by exploring these spaces of action possibilities, thereby acquiring expertise how to interact with the world expediently. They develop a style of interacting, and certain ways of interacting become entrenched not only because they are physically expedient, but also because they are culturally expedient. People do not consider (and have never considered) the full range of action possibilities open to them. They do what has become familiar to them and what is familiar from observing other people. Nevertheless, they acquire their own repertoire of specific ways of interacting with the world and thereby they cultivate their own experience of interactions with the world. They establish their own perspective rooted in their sensorimotor expertise—some more commonly shared between people, some highly specific to individuals. One can illustrate this on the example of a cup. A cup is graspable, but there are many possibilities how to grasp it. For example, a person could ignore the cup’s handle and grab the container itself, grasp the container while slipping a finger through the handle, or grab the handle without touching the container. As humans are surrounded by cups all the time, they usually have a considerable repertoire of expertise for how to interact. There are additional contextual constraints in which ways of interacting are more expedient, e.g., the cup’s content and its capacity to insulate that content. Some agents may tend to hold cups by slipping a finger though the handle, presumably because it is a very secure and stable way of grasping a cup. But if a cup is filled with scalding coffee, this may expose the agent’s hand to uncomfortable levels of heat. A regular coffee drinker will have experienced this often and will perceive their cup of coffee to be graspable only by its handle.

### Contextual Dependence of Embodied Moral Judgments

The assertion of this paper is that this sort of sensorimotor expertise should be understood as a vital component of human moral interactions. People experience the permissibility of their actions depending on their specific repertoire of sensorimotor expertise. The embodied judgments characterized in the last section, i.e., styles of learned motor-patterns, can be understood as an acquired sensorimotor expertise.

Let us turn to the second claim VTR make about embodied action: “individual sensorimotor capacities are themselves embedded in a more encompassing biological, psychological, and cultural context.” Understanding this second claim is integral to understanding the flexibility of moral experience. It feels wrong to touch a stranger on the street, but right to touch your partner in your home. The example of the cup is instructive once again to exemplify the individuation of experience relative to different contexts. Imagine you must carry a cup of coffee for a short distance. In this situation, the threat of spilling may be more prominent than the temperature of the container. In this case, it is expedient to grasp the container rather than the handle. But agents also have a personal style in doing so. A server may have sufficient trust in their ability to transport a cup by its handle without spilling. Someone who has badly burned themselves by spilling may never prefer the handle, so as to minimize the chance of spillage. These are not conscious, reflected decisions: Agents usually just grab the cup and, because of their acquired expertise, grab it in a way appropriate to the context. If they misinterpret the context, e.g., the cup being hot, they will adjust fluidly and automatically to other ways of interacting, taking into account the newly perceived context. This process unfolds dynamically: a grasping motion is adjusted, as steam emanating from the cup is perceived, because the experience of how the cup is graspable shifts.

Streuber (2013) asked participants to complete a simple sensorimotor task in a virtual environment. Participants either high-fived a human-looking avatar or a robotic arm. The movement of the arm, whether it was a robotic or a human-looking avatar, was precisely the same in every trial. The participants, however, performed very different movements with their arm, which were tracked using a sophisticated motion capture method. If they high-fived a human, they performed a fluid motion, much like one would expect from everyday life. But with the robot arm, people change their style. They performed a rather box-like movement, raising their hand to an appropriate height and then moving it forward to make contact. This is interesting because people are not relying on the same expertise to perform the movement in one context as they do in the other context. Context is not defined by the physical properties of the environment; otherwise, the uniformity in movement of the avatars would result in participants utilizing the same expertise. The difference is in how the avatar is perceived, either as a human or a robot. The participants relate to these avatars differently, perceiving humans as high-fivable in one way and robots as high-fivable in a different way. This goes a long way to resolve the problem of flexibility. Similar actions, like touching a stranger vs. touching one’s partner, are not similar at all, if we relate to the people involved differently. People need not rely on more than their experience to act appropriately in most situations, as long as they relate appropriately to others. Their relationship specifies which actions are perceived as appropriate.

These broad differences in relating to the world are only the tip of the iceberg. People make all sorts of differentiations between each other and most are morally unproblematic, e.g., when one relates to others as friends, family, partners, co-workers, neighbors, and so forth. Moral experiences are often specific to these relationships. For instance, hugging a co-worker may feel inappropriate, especially compared to the experience of hugging one’s friends. Additionally, *how* a hug is carried out might be experienced as more or less appropriate depending on the (relational and situational) context. Packheiser et al. (2018) present evidence that motor lateralization in hugging, i.e., whether you hug someone toward the left or right, depends on the emotional context. People have a preference for right-sided hugs, but there is a statistically significant difference between the relational context. People embracing in an airport do so on the right side in about 81% of cases, while strangers hugging in YouTube videos do so in about 92% of cases. We infer that there is a relationship component, because it seems likely that a hug in an airport implies some sort of relationship, while the YouTube data are explicitly selected for unfamiliarity.^7^ More definitive evidence is given in a kissing study by Sedgewick and Elias (2016), who analyze pictures of parental and romantic kissing. They find that people tend to tilt their head to the right in romantic kissing, but to the left in parental kissing. We consider this further evidence for the thesis that sensorimotor expertise is relationally specific, i.e., that the movements a person considers, experiences as appropriate, and is motivated to carry out depend on the expertise and previous experience of actions in that relationship.

At this point, we have to briefly compare notes with a few ethicists. Can their claim be understood in terms of the cognitive considerations advanced so far? Nussbaum (1995), following many other eminent feminist writers, decries the practice of objectification, i.e., relating to another human as an object. To treat someone as an object is to not perceive the things as wrong that would be perceived as wrong when relating to someone as a subject. Another related concept can be found in the practice of Othering (cf. Levinas, 1969), where the “face” of another human being is denied, i.e., one does not relate to them as an other but as the other. Levinas’s phenomenology is hard to penetrate, but the salient point is the same: Relating to another person as a non-person is one of the principal moral failings—one fails to perceive their humanity, vulnerability, and dependence. Both of these claims are easily accounted for. Our moral experiences have to rely on the sensorimotor expertise acquired in the right relationships, i.e., with persons (in Nussbaum’s case) and others (in Levinas’s case). If one engages with another person as if they were an object, one experiences this interaction as if it were with an object and not a person. Thus, an agent in such an interaction will lack the appropriate moral experiences, because the expertise underlying these experiences was acquired through interacting with objects and not other humans. Pulling a lever is not experienced as impermissible, because there is a lack of previous experiences which rendered this action possibility impermissible. Pushing a person, however, is a person-specific action possibility, and, thus, is experienced negatively because of the previous experiences with other people. Pushing another human is not perceived as permissible because one has previously empathized with their pain or was subject to social resentment for such actions. Extending a cognitive account of moral decision-making to encompass these moral phenomena is no small feat. After all, Virginia Held (1996, 72–75) rejected the claim that cognitive science had anything to offer ethics because of its inability to account for moral experiences.

### Layered Relational Context

**FIGURE 2:**
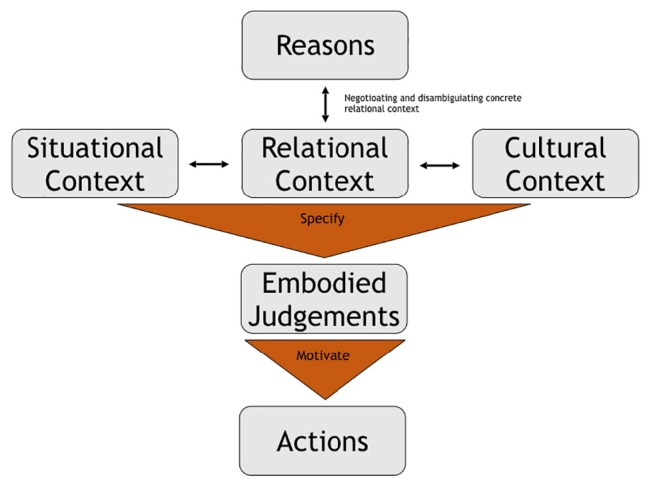
Embodied account of moral action.

So far we have established that some moral judgments are embodied and relationally embedded, thus being flexible enough to account for the variety of human moral judgments. This flexibility is only one side of the coin, as agents in concrete situations require a concrete relational context to make these situations intelligible to them. A friend of a germaphobe, for example, will neither hug nor shake the other’s hand and instead opt for more hygienic forms of greeting and farewell within this concrete relationship. The rich variety of normative structures enacted in interpersonal relationships is not captured by bland generalizations. There are often overlapping layers of relational context, each with their own normative structures. Societies have broad normative structures in place regulating how to relate to specific people and what is appropriate in the relationships we share with them. It may be an appropriate form of greeting to kiss a woman on the cheek, while men shake hands, etc. This is often modified by concrete situational context; it is not appropriate to grab someone on the street, for example, but if they are in danger of running in front of a car, it is permissible (maybe even required) to grab them in order to protect them. When someone is in danger, there is situational context overriding the general cultural norms (Figure 2).

The most interesting layer of relational context is that of concrete relationships established by the interactions of moral agents. It is within the interactions between agents that the (in)appropriateness of certain ways of interacting is established. Consider the case of Alex and Drew who just met in a casual setting and are now trying to form a connection with each other (compare McGann and De Jaegher, 2009, who discuss a similar case in the enactive framework). Of course, they do not start from scratch when beginning to build their relationship. Through the experiences they have made in previous relationships and through observing the relationships of others around them, both will have acquired a repertoire of relational “blueprints” which provide them with prototypical normative structures for a certain relationship type (e.g., stranger, co-worker, friend, therapist, teacher). If relationships develop and agents grow closer, however, the normative structures adopted from the blueprints will often be altered and individualized through the way the agents interact with each other. While many such adjustments are made across longer periods of time, the very beginning of a relationship often includes rapid changes. Partially, this is because agents learn about many personal preferences that may contradict the relevant relational blueprint. Suppose, for instance, that Alex is attentive and learns that Drew loathes small talk. Given that Alex cares about establishing and maintaining a (good) relationship with Drew, small talk will quickly come to be seen as something inappropriate for this particular relationship, even if Alex generally considers it to be permissible or even polite.

The other reason for the rapid changes in what is considered appropriate after agents first meet is that the relationship they enter into will often need to be disambiguated. There are, of course, some cases in which it is clear to both agents what kind of relationship they are building and what they can—in all probability—expect from it. This will be true, for example, of most teacher–student and therapist–patient relationships. In other cases, however, the relational structure will be less clear. Alex and Drew, for instance, might initially be unsure of how to understand the nature of their interaction: what registers as flirting to Drew might only be considered friendly conversation by Alex. Even if they both eventually come to see their interaction as flirting, it still remains unclear whether it might lead to a (single) sexual encounter, build the foundation for future romantic encounters, or find its conclusion when they go their separate ways. Within their interaction, then, Alex and Drew must disambiguate the situation and try to jointly establish a clear relational context. Such a disambiguation can, of course, happen in conversation where one of the agents simply asks the other how they understand their relationship. Often, however, this kind of conversation does not arise and, even if it did, the agents might not yet have discerned what they expect from the relationship to a sufficient degree to have a conversation about it. Instead, agents will often disambiguate their relationship through acting, reacting, and adjusting. Suppose that Alex puts a hand on Drew’s thigh. Both of them understand this kind of physical contact as sexual and, therefore, Alex’s hand placement as an attempt to establish a sexual component in their relationship. If Drew reacts positively and responds in kind, further sexual advances will be considered permissible. However, if Drew grows visibly uncomfortable, Alex will ideally understand this discomfort to indicate Drew’s unwillingness to add a sexual component to their relationship (at this moment). Which ways of interacting with Drew Alex considers appropriate or inappropriate, then, is shaped by Drew’s actions and reactions. Within this interaction, then, sexual touches acquire an evaluative-affective component for this concrete relationship similar to how the motor patterns of pushing someone have acquired a negative one in social learning interactions. Embodied judgments, then, can be extremely flexible in accounting not just for why participants choose not to push the large person but also for why agents choose to hug or shake hands and why kissing is deemed an appropriate interaction with one person but not another. Thus, the relevance of embodied moral judgments potentially extends beyond personal force factors to a multitude of interpersonal interactions.

## Implications for Culture and Moral Philosophy

Thus far, we have argued for the embodied (and pre-reflective) nature of judgments. But it is important to look at the broader implications of such a position. Doubting the causal efficacy of reasons brings up a number of issues: From the their role in moral practices of Western culture, to the understanding of morality itself. Why do we frequently appeal to reasons in everyday life, if they have often very little to do with our action? How can moral philosophy discuss moral questions, if there are no reasons to analyze or if there are no moral agents that can act according to the tenants of a particular moral theory?

We will first explore why cultural practices relying on “reason talk” can still be meaningful. While it is often assumed that “reason talk” gives morally relevant information by expressing causative motivating forces for action, we believe that such talk is a tool to communicate relational information. This relational information is indirectly action relevant, as embodied judgments depend on the context of a situation, a context that can be shaped by interactors through expressing reason. This provides the basis to understand what an embodied moral theory might look like. Rather than analyzing reasons for action, an embodied moral theory analyzes how specific moral judgments were cultivated and whether the moral context they are situated in is appropriate. An embodied moral theory does not look at a problematic action in isolation, but rather considers the developmental, relational, and situational factors. We may, for example, look at the narratives that shape a particular relationship of an agent with the world to determine whether such a narrative provides a suitable basis to form appropriate moral judgments. In turn, this may enable ethicists to articulate standards for cultivating embodied judgments in particular contexts and how to relate to the world such that these relationships are conducive to appropriate actions. But first, we will defend the position that “reason talk” is still morally significant as a cultural practice, even though it may be often misunderstood.

### Are Moral Practices Meaningless?

If reason is just a post-facto confabulation, not reflecting the true causative motivational forces of actions, then much of the social discourse centered around the reasons an agent has for acting, or attempting to convince agents to reflectively endorse other reasons, makes very little sense. When an agent explains their reasons to a friend who was angered by their actions, for example, this is often experienced as an accurate presentation of what lead to the action in question. In giving this explanation, the agent tries to make their friend see how their action was the (inevitable) product of their reasoning process. However, if the reasons that the agent presents had no part in bringing about the action in question, articulating these reasons seems to be meaningless. Which reasons are articulated, then, should not have an effect on the anger the agent’s friend feels. In this section, we will argue that even though the agent’s explanation of what motivated them is inaccurate in most cases, it is still a meaningful practice of shaping a moral community. It is important to note here that the practice of appealing to reason as a culturally specific to Anglo-Europeans (Lillard, 1997). It is, thus, not a prerequisite for moral practices in a society, it is just one way to articulate morally relevant information.

To investigate how the articulation of reasons can be meaningful even though the judgments they are supposedly explaining are embodied, i.e., lacking amodal, propositional representational content, we return to the first meeting of Alex and Drew depicted above. Suppose that Drew reacted negatively and Alex recognized this negative reaction. So their relationship does not allow for any sexual components. Alex now perceives touching Drew in a sexual way as impermissible. Qua our theory, this is a consequence of the relationship structure enacted in which these actions now have a negative affective evaluative experience. But Alex can still articulate reasons for not taking these actions now, e.g., that Alex does not want to risk their friendship. We have assumed in the beginning that these reasons are probably not effective in guiding Alex’s actions. So is Alex’s reflective access to these reasons and their articulation in an interpersonal context just a meaningless exercise of a human language game? On the contrary: it appears that in giving an explanation, inaccurate as it may be, Alex expresses a desire as to how the relationship with Drew should develop in the future. This becomes especially clear in those situations in which relationships might change significantly. Consider what would happen, for example, if Alex and Drew did have sex with each other after having been friends for a while. When the two of them sit down to discuss why they slept with each other and where to go from there, Alex gives one of the following explanations: (1) “I had sex with you because I have romantic feelings for you,” (2) “We’re both single, we like each other, I thought we could have some casual fun,” or (3) “I was drunk and lonely, this was a mistake.” Those statements are ostensibly statements of motivation for the action in question and in this sense unlikely to be accurate. However, even if that is the case, the explanation Alex offers is still meaningful because this explanation will become a narrative element in the ongoing process of relationship-shaping. While the first explanation expresses a clear desire to re-shape the relationship with Drew from a platonic friendship into a romantic relationship, the second explanation expresses the desire to add a sexual component to the friendship and the third explanation conveys the wish not to re-shape the relationship at all.

The moral practice of articulating reasons, then, is still meaningful as it is an important narrative tool to clarify relational context, in which embodied actions are embedded. People tell each other (and themselves) stories about their lives and relationships, that need to clarify the significance of particular actions within them. To express a reason is not at all to make a factual statement. Rather people engage in a sort of mental fictionalism, which is useful and convenient to them as a shared currency to negotiate their lives. This does not mean, of course, that all narratives are equally permissible. After all, it is easy to construct situations in which a narrative is told in order to maliciously manipulate another’s perception of a situation. In the following, we will discuss normative demands that might be applied to how relationships and interactions are narrated.

Narrative approaches feature both in ethics and cognitive science, though in both they are far from dominant. However, as we attempt to explicate a cognitive practice with moral content, this overlap is more than fortunate. We make two claims here: (i) that our articulation of reasons is useful to create a shared moral space, a primordial moral community, and (ii) that narratives are instrumental in understanding the moral landscapes of our lives.

The first claim concerns the problem of other minds, i.e., how people understand each other and know what they think. Rationalist approaches like theory, folk psychology, or simulation theory are considered unconvincing nowadays and are being modified to remedy their shortcomings. Hutto (2007), for example, put forth his narrative practice account: “[I]t is through direct encounters with stories about reasons for acting, those supplied by responsive caregivers in interactive contexts, that children become familiar with (i) the core structure of folk psychology and (ii) the norm-governed possibilities for wielding it in practice” (Hutto, 2007, 117). In his account, understanding others by expressing reasons is not a matter of fiat, as “reason-based understandings are not used by our close living cousins, the chimpanzees, nor were they used by our ancient ancestors who hailed from the Pleistocene” (Hutto, 2007, 116). It is also not necessarily the only way in which people understand each other. Rather, the creation of narratives is a specialized tool because “narratives function as ‘normalizing’ explanations, allowing us to cope with ‘unusual’ or ‘eccentric’ actions, where possible, by putting them in context” (Hutto, 2007, 119). This is exactly what people do in their moral practices when they appeal to reason-based explanations: They try to explain moral actions which require explanation, not those which are understandable to all involved to begin with. Moral communities are built on shared moral understanding; moral discourse, however, is usually centered on disagreement. So narratives occupy a central place in moral discourse because they allow for moral disagreements to be negotiated.

MacIntyre advocates a broader account of interwoven historical narratives that give rise to the identity of a person, as well as a community. MacIntyre emphasizes the importance of social structure because “the unity of a human life becomes invisible to us when a sharp separation is made either between the individual and the roles that he or she plays” in society (MacIntyre, 1981, 204). According to him, the modernist preoccupation with singular actions renders our moral knowledge inaccessible. “For a self separated from its roles […] loses that arena of social relationships in which the Aristotelian virtues function if they function at all” (MacIntyre, 1981, 205). MacIntyre’s insights are echoed in our ruminations on the embodied nature of judgments, which likewise have to be understood in the context of the relationships they are cultivated in. However, we are somewhat skeptical of an Aristotelian, neo-Aristotelian, or Thomist interpretation of these insights^8^.

### Non-Rational Agency and Relational Ethics

The much criticized Cartesian spirit pervading ethics and cognitive science often manifests in an unease about non-rational agency. If we have no or little reflective reason-based control over outraction, how could we be considered moral agents? We, however, believe this to be an issue born from the Cartesian mindset and not a substantive philosophical issue. Both in cognitive science and ethics, many writers have recognized that agency can also be understood in non-Cartesian terms. On the cognitive side of the debate, we have Varela et al. (1991, p. 106–122) who rather than give up the idea of agency in the face of embodied pre-reflective cognitive processes, propose a unitary theory of mind that includes these processes as constitutive processes of personal agency (which has to include moral agency. We have dealt with the issue of enactive theory and moral agency elsewhere, authors, and thus will not go any further here.) In ethical discourse, we can draw, in addition to MacIntyre’s contributions mentioned earlier, on the late work of Ricoeur (1984, 1985, 1988) to appreciate that agency may be understood in non-Cartesian terms. Ricœur’s analysis of narrative identity lines up well with our own considerations concerning moral agency in the enactive tradition and the following analysis of narratives as they relate to their role in shaping relational context.

Narratives are crucial in understanding each other, oneself, one’s life, and therefore one’s moral community. It is thus our contention that the correct unit of analysis of moral questions is not an individual agent’s reflected motives, intentions, or reasons for an act viewed in isolation. A cognitively adequate ethical analysis has to focus on the appropriateness of a judgment in a relational context and the appropriateness of the relational context established. The most explicit way in which these relational contexts are articulated are the narratives accompanying them, and thus we will provide a tentative exploration of ethical dimensions of narratives. We will focus on the question of appropriateness of the relational context, rather than exploring the question of cultivation of appropriate judgments.

### Moral Standards for Narratives and Coherence

Experiences of normativity are not always appropriate or justified. One of the corollaries of the position we have been advancing in this paper is that analysis of these concrete normative questions should be focused on the narratives (or more broadly the relational context) moral experiences are embedded in. Before, we argued extensively for the flexibility of relational contexts, especially as they are aided by narratives. However, narratives can, and historically often have, aided not only virtuous endeavors, but often justified horrific crimes. A moral transgressor often offers a narrative of their actions, which in their eyes justifies their actions. In other words, narratives are *too* flexible. Not all the experiences that moral agents can conjure through committing to different narratives are experiences that moral agents should have. Ethical analysis can put moral standards in place specifying which narratives an agent should commit to.

The first issues we take up are insular moral narratives: those that are not embedded in a broader context of socio-cultural and interpersonal moral narratives, but are separate from them and, thus, express relational structures, which might be impermissible if viewed in context. It is common that what is permissible in a concrete relationship is impermissible in society at large, but usually a society’s norms are still compatible with the ones enacted in the concrete relationship (e.g., romantic kissing is not a socially permissible greeting, but it is socially permissible to enact a relationship in which kissing is a permissible greeting). Contrary to this, a therapist and one of their patients may enact a relational structure that renders sex permissible, but this is not compatible with the socio-cultural context. In enacting their relationship as they do, the therapist and their patient create a narrative that lacks broader coherence with social values. In other cases, narratives are constructed that are actually incompatible with the interpersonal relationship they pertain to: In a concrete relationship, we may find that one person claims sole ownership of the relational narrative even in contradiction to the other party’s explicit statements. Drew, from the earlier example, after being told by Alex that their night together was a mistake, may insist that their relationship is now a romantic relationship, ignoring Alex’s statements to the contrary. If Drew really commits to this insular narrative, then Drew may experience that it would be permissible to touch or kiss Alex. But these experiences are easily revealed as inappropriate by analyzing the relational context and revealing it to be incoherent, and thus falling short of one possible moral standard we may hold this narrative to.

To place importance on coherence may be problematic because it may render moral communities and personal relations static and unable to change: A stable relationship may appear to become more incoherent, when attempts to change it are made, but often changing relationships is a good thing. Moral changes are aimed at bringing socio-cultural and interpersonal narratives into more coherence, not less. For example extending rights and privileges to women was never to make social structures incoherent—even though it may have been portrayed in this way. Rather, it resolved the incoherences produced by separating people into groups that differed in status. Similarly, changing a friendship to a romantic relationship is permissible if (and only if) this is in the involved persons’ interests and with their consent.

But how do people know whether a specific differentiation in relational context is permissible or not? To Drew it is apparently not obvious that the romantic relational context is rendering the narrative more and not less incoherent. Why would it be morally questionable to pursue establishing such a narrative? We have ourselves argued that the appeal to reason in narratives, though inaccurate, is often not harmful and convenient. As MacIntyre (1981, p. xx) points out, narratives form complicated webs of personal, interpersonal, communal, and cultural narratives. These narratives do not exist for their own sake, but are tested in the practices and actions of the individuals of each moral community. Alex and Drew did test a different relational structure, but they experienced it very differently. To test narratives is often necessary to figure out whether they are actually the ones that fit a concrete relationship. What is problematic here is that Alex’s experience of this situation is cast aside, even though Alex is one of the parties directly involved and the norms enacted in this relationship directly pertain to Alex.

#### Majority Narratives, Power, and Force

Moral agents in a moral community should have a voice in it; they should be empowered to speak, be heard and not marginalized for doing so. However, this basic right is not always afforded to all people equally, e.g., women, peasants in the middle-ages, and workers since the industrial revolution. This occurs in part because power can be turned into moral narratives. The prince can pay Machiavelli to justify his personal experience, by providing a strong narrative of his exceptional position in life. (White, heterosexual, middle-class) Men, as sole custodians of the moral narratives of Western civilization, naturally expressed *their* experience of the moral practices. The problem is that the impact of moral practices is not felt equally by all people in a moral community. It is unsurprising that practices that affect those without a voice persist even though they are clearly incoherent from their point of view. It is obvious to Alex that Drew’s attempts at establishing a romantic relationship is changing their relationship for the worse, but Drew is unaware of this. If Alex has a voice and is allowed to shape the relational narrative, this does not become problematic. But if Drew is in a position to dictate the narrative, the narrative can easily become morally problematic.

The remedy to this can be derived from Hutto’s work on narratives emphasizing their interpersonal role in facilitating mutual intelligibility. Power can be a hindrance to such mutual intelligibility. As power turns into force, the perspective of a less powerful person is easily dismissed. They have to live with a narrative imposed on them, though it makes very little sense to them. The remedy is thus that moral communities embrace moral practices that are jointly enacted by the people they pertain to, and to empower these people to test these narratives, so that all people’s voices are being heard. A moral community thus should be built such that the experiences of its members are intelligible and accessible to each other. But how can people share the experiences of others?

#### Joint and Caring Narratives

The theory we favor to explain how people gain mutual understanding, i.e., to enact normative contexts together, is participatory sense-making (De Jaegher and Di Paolo, 2007). This theory is an expansion of the enactive account into social cognition, which naturally fits with the embodied account of judgment provided here. Participatory sense-making emphasizes the importance of interaction for mutual understanding. Interactors are sensitive and reactive to each other, forming a coupled system. This coupled system does not infringe on their individual agency. As it is constituted by both interactors’ agency, it becomes the center of a perspective both interactors are party to. Through the interaction, agents negotiate their perspective on life (and, thus, the relational structures they enact) not merely from their own limited perspectives, but rather from a joint perspective. Colombetti and Torrance (2009) picked up on the potential to enrich ethical discourse with this concept. Urban (2015a, b, 2016) provides critical conceptual analysis to link this idea to a major branch of contemporary ethics: care ethics, which emphasizes the importance of good (i.e., caring) relationships. But care ethics is equally sensitive to the trust involved in a relationship, which cannot be reduced to the perspective of an individual (Held, 2006).

As the currency of discourse in a moral community are the narratives embraced by its members, it is natural to analyze these narratives in terms of the interactions that produced them. If these interactions were caring, the narrative produced jointly enacted by its participants, then it is a normatively superior narrative than those created in isolation, by force or paternalism.

## Conclusion

In this paper, we set out to understand the nature of moral judgments. The orthodox view is that reasons, i.e., propositional, amodal, reflected beliefs, inform judgments which motivate behavior, making it plausible to appeal to these reasons in moral discourse: “Why did you do X?” “Because of reason Y!” Moral philosophy, in turn, is often concerned with an analysis of reasons in this spirit. However, we believe that there is sufficient evidence to doubt that this picture is really accurate. We set out to explore alternatives to the orthodox reason-based account of pre-reflective judgment, mindful not to forget that the common moral practice of reasons has to fit into this picture somehow, if not in the privileged place afforded to it in orthodox theories.

Evidence from cognitive science and moral cognition research suggests that agents form pre-reflective judgments that are embodied in nature, which motivate their actions. These judgments are particular to the relationships an agent enacts with the world and particular to the interpersonal relationships an agent engages in. Because these judgments are embedded in relational structures, they are more flexible than commonly thought, and we can, thus, use this account to explain the variety and flexibility of human moral judgments and behaviors. We do not need to rely on reason-based conception of judgments in these explanations.

Still, this reflective type of judgment may occasionally be action relevant. The primary function of reason-based practices in moral communities, however, is not to transmit knowledge to motivate good behavior. In Anglo-European cultures, these practices are dominant and the efficacy of reasons has been somewhat overstated. In our view, these practices have the simple function of allowing discourse on relational uncertainty. Reasons are articulated to clarify relational narratives. Narratives are culturally idiosyncratic, and reasons are useful as narrative elements for people who are accustomed to such a practice. Articulating reasons helps people to overcome breakdowns and unclarity in relational contexts by clarifying the narrative structures accompanying the relationships or shaping a relational structure for the future by changing its narrative structure.

It has been noted that it is ironic or contradictory that we present reasons to doubt the efficacy of reasons. Rather than this being a self-defeating argument, the realm our claims pertain to and the realm in which we make these claims are not the same. In the scientific and philosophical context we utter these claims the reason-based language game we are still committed to makes a lot of sense. In a community that has cultivated their use and understanding of language in a specific way, it is an expedient way to communicate insights. However, just because the audience this text is intended for has spent decades of their lives cultivating their minds to make sense of this language game, does not mean that this way of communicating is to be styled into a way of life. Ethicists, even though skilled at the language games of moral philosophy, are not better people. Their reflected knowledge does not translate into good actions, a fact that in itself lends credence to the analysis advanced in this paper.

## Author Contributions

All authors listed have made a substantial, direct and intellectual contribution to the work, and approved it for publication.

## Conflict of Interest

The authors declare that the research was conducted in the absence of any commercial or financial relationships that could be construed as a potential conflict of interest.

## Footnotes

1 The replicability of this study has been doubted. We present it anyway because the experiment deals with a genuinely moral question and studies by Darley and Latané (1968); Latané and Rodin (1969), and Latané and Dabbs (1975) show similar effects in other circumstances.

2 Schroeder makes reference to the orbito-frontal cortex, a different name for the ventromedial prefrontal cortex. We opt to refer to the brain area in question by “vmPFC” to have consistent terminology with Joshua Greene’s work.

3 68.2% of professional philosophers indicate this to be the choice they would endorse (Bourget and Chalmers, 2014).

4 Levinas’s phenomenological (meta-)ethics especially seem applicable to explain this problem, after all, he writes (1969, p.199) “The impossibility of killing does not have a simple negative and formal signification: [It comes about] as a face in the ethical resistance that paralyzes my powers […]” For Levinas the recognition of another as a moral agent, one that ought not to be killed, comes from our recognition of their face. The bodily contact with another human will reveal such a face, especially if this interaction recognizes their vulnerability. A button or a lever may hide the face of the affected person, but a direct physical interaction that would exploit another human’s vulnerability, almost necessarily reveals their face.

5 Men are equally but differently affected by the cultural norms of bodily style. Professional basketball players for example still refuse to deliver a free-throw in the most effective way (Venkadesan and Mahadevan, 2017), though they usually are pretty committed to enhancing their performance. The problem is that the most effective way to deliver a free-throw is to do it “granny-style,” i.e., to swing the ball with both hands before one’s body and deliver it like this.

6 Compare Colombetti and Krueger’s (2015, 1069) application of a similar line of thought to the extended mind view. The concept of scaffolding may provide a useful alternative basis for a non-orthodox account of the moral mind.

7 The assertions made by Packheiser et al. that hugging a stranger is emotionally neutral, while hugging a person in an airport is not, is questionable. Getting accosted by a YouTuber to be hugged will probably entail some emotional reaction.

8 It is possible to cast our account of judgment into a virtue ethics framework, as it shares many of their preoccupations. One may consider it a virtue for people to cultivate the right kinds of judgments in the right kinds of relationships. However, we are cognizant of the situationist troubles character-trait-based accounts of virtues find themselves in, which we certainly avoid with our approach. However, it would not be a novelty to set aside character traits and provide a more psychologically informed account of virtues (e.g., Snow, 2010; Miller, 2014).
